# Evaluation of a Collaborative Protocolized Approach by Community Pharmacists and General Medical Practitioners for an Australian Minor Ailments Scheme: Protocol for a Cluster Randomized Controlled Trial

**DOI:** 10.2196/13973

**Published:** 2019-08-09

**Authors:** Sarah Dineen-Griffin, Victoria Garcia-Cardenas, Kris Rogers, Kylie Williams, Shalom Isaac Benrimoj

**Affiliations:** 1 Graduate School of Health University of Technology Sydney Ultimo Australia; 2 Emeritus Professor The University of Sydney Camperdown Australia

**Keywords:** pharmacy, pharmacists, general practitioners, primary health care, community pharmacy services, nonprescription drugs, self care, self medication, randomized controlled trial, Australia

## Abstract

**Background:**

Internationally, governments have been investing in supporting pharmacists to take on an expanded role to support self-care for health system efficiency. There is consistent evidence that minor ailment schemes (MASs) promote efficiencies within the health care system. The cost savings and health outcomes demonstrated in the United Kingdom and Canada open up new opportunities for pharmacists to effect sustainable changes through MAS delivery in Australia.

**Objective:**

This trial aims to evaluate the clinical, economic, and humanistic impact of an Australian Minor Ailments Service (AMAS) compared with usual pharmacy care in a cluster randomized controlled trial (cRCT) in Western Sydney, Australia.

**Methods:**

The cRCT design has an intervention group and a control group, comparing individuals receiving a structured intervention (AMAS) with those receiving usual care for specific health ailments. Participants will be community pharmacies, general practices, and patients located in Western Sydney Primary Health Network (WSPHN) region. A total of 30 community pharmacies will be randomly assigned to either intervention or control group. Each will recruit 24 patients, aged 18 years or older, presenting to the pharmacy in person with a symptom-based or product-based request for one of the following ailments: reflux, cough, common cold, headache (tension or migraine), primary dysmenorrhea, or low back pain. Intervention pharmacists will deliver protocolized care to patients using clinical treatment pathways with agreed referral points and collaborative systems boosting clinician-pharmacist communication. Patients recruited in control pharmacies will receive usual care. The coprimary outcomes are rates of appropriate recommendation of nonprescription medicines and rates of appropriate medical referral. Secondary outcomes include self-reported symptom resolution, health services resource utilization, and EuroQoL Visual Analogue Scale. Differences in primary outcomes between groups will be analyzed at the individual patient level accounting for correlation within clusters with generalized estimating equations. The economic impact of the model will be evaluated by cost-utility and cost-effectiveness analysis compared with usual care.

**Results:**

The study began in July 2018. Thirty community pharmacies were recruited. Pharmacists from the 15 intervention pharmacies were trained. A total of 27 general practices consented. Pharmacy patient recruitment began in August 2018 and was completed on March 31, 2019.

**Conclusions:**

This study may demonstrate the efficacy of a protocolized intervention to manage minor ailments in the community and will assess the clinical, economic, and humanistic impact of this intervention in Australian pharmacy practice. Pharmacists supporting patient self-care and appropriate self-medication may contribute to greater efficiency of health care resources and integration of self-care in the health system. The proposed model and developed educational content may form the basis of a national MAS service in Australia, using a robust framework for management and referral for common ailments.

**Trial Registration:**

Australian New Zealand Clinical Trials Registry (ANZCTR) ACTRN12618000286246; http://www.anzctr.org.au/ACTRN12618000286246.aspx

**International Registered Report Identifier (IRRID):**

DERR1-10.2196/13973

## Introduction

Integrated care is a possible solution to the rising demand in facilitating appropriate delivery of health services and limiting fragmentation between health care providers. Evidence indicates that health systems with strong integrated primary health care are effective in improving patient outcomes and efficient at delivering high-quality appropriate services [1,2]. Many countries have undergone major health reforms to deliver effective and efficient health care, moving toward sustainable health systems that are both durable and resilient to withstand impending and ongoing challenges [3-6]. As an example, the Australian health system has undertaken significant reform and restructuring to improve value for investment in health care [2,7] through the establishment of Primary Health Networks (PHNs). Their objectives are delineated as (1) delivering health care services that increase the efficiency and effectiveness for patients and (2) strengthening the degree of coordination and connectivity of care, ensuring patients receive the right care, in the right place, at the right time [8].

Major questions exist surrounding how health care systems can address minor ailments more efficiently through the use of administering care in less expensive settings such as community pharmacy [9,10]. Minor ailments have been defined as “conditions that are often self-limiting, with symptoms easily recognized and described by the patient and falling within the scope of pharmacist’s knowledge and training to treat” [11]. It is already known that patients self-manage conditions to a large extent [12], and encouraging people to exercise greater levels of self-care, either for acute or chronic problems, has significant potential to directly affect demand for, and shift costs from, medical health care. Pharmacists are positioned to facilitate self-care and appropriate self-medication processes [13]. Undoubtedly, the expansion of nonprescription medicines has given patients greater choice, providing community pharmacy with an opportunity to demonstrate real and tangible benefits by facilitating this process [13]. Community pharmacy has been transforming to a service provider model driven primarily by leadership of professional organizations, government policies, remuneration, and patient needs. The community pharmacy sector has undergone changes such as enhancing the pharmacists’ role in providing professional pharmacy services to optimize the process of care [14]. Community pharmacy provides a range of remunerated commissioned and noncommissioned professional pharmacy services that have shown to be cost-effective compared with other health care settings and contribute to improved health outcomes for patients [15-18]. Importantly, pharmacists can be better integrated within primary care. Effective collaboration between general medical teams and community pharmacies will be integral to achieve the highest level of patient care [8,19].

There is consistent evidence at an international level that pharmacy-based minor ailment schemes (MASs) promote efficiencies of use within the health care system [20]. MASs were introduced for patients to access professional support for conditions that can be self-managed with the objectives of increasing accessibility, providing the right level of care and mitigate funding and system inefficiencies [21]. A total of 94 international schemes are identified in the literature across 103 regions, including the United Kingdom (England, Scotland, Northern Ireland, and Wales) [20,22-26]. Minor ailment assessment and prescribing is the nomenclature used in Canada, representing a pharmacy service that allows pharmacists to prescribe certain drug groups for the treatment of minor, self-diagnosed, and/or self-limiting conditions. Of 13 provinces in Canada, 8 operate a Minor Ailments Prescribing Service [27-28]. Each of these services is slightly unique in its feature and structural design parameters [20]. MASs have been included in the policy agenda in Australia [29-31] and New Zealand [32]. Paudyal et al explored the effect of MAS on patient health and cost-related outcomes [21]. The review showed low reconsultation and high symptom resolution rates of up to 94% with MAS, suggesting minor ailments are being dealt with appropriately in pharmacy [21]. The positive economic impact has shown international MAS to be cost-effective compared with more expensive health care services, such as general practice and accident and emergency (A&E) departments [16]. There are different models of general practitioner (GP)-pharmacist collaboration offering the community pharmacy network to be better integrated into general practice or urgent and emergency care systems. One example in the United Kingdom is the provision of integrated out-of-hours services by community pharmacy, such as the Digital Minor Illness Referral Service [12]. The service evaluates the way in which patients with self-limiting minor ailments who are contacting urgent services can be supported by community pharmacists instead of being booked for an urgent GP appointment or signposted to their own GP.

Pharmacists treating patient’s common ailments, the exclusive availability of nonprescription products through pharmacies to provide symptomatic relief, and referral to other health care professionals is a well-established activity within pharmacy practice. Unfortunately, in Australia, there is limited standardization and protocolization for consultations and procedures for escalating referral. There is minimal integration with general practice systems and no formal method of physician-pharmacist collaboration or communication relating to minor ailments, and the nature and extent of collaboration may be seen as both episodic and informal. This invariably limits facilitated self-medication practices. In addition, there are no mechanisms to monitor or document patient interactions, resulting in missed opportunities to identify patients who require referral, limiting the ability to detect inappropriate or continued use of nonprescription medicines. The potential for community pharmacists to moderate patients’ needs for the treatment and management of minor ailments and alleviate health system pressure in Australia has been recognized [33,34].

The Australian Minor Ailments Service (AMAS) is a practice model with key elements, such as agreed referral points, communication systems between pharmacists and general practitioners (GPs), and clinical treatment pathways, that is, *HealthPathways*. The conceptualized components of AMAS have been developed in consultation with key stakeholders including PHN leaders and, importantly, leading general medical professionals involved in PHN governance in Australia. Input into design and agreement with stakeholders have progressed the development of collaborative referral pathways, providing a robust framework for community pharmacists to deliver evidence-based minor ailment care. In essence, these pathways seek to improve the coordination and delineation of health care provider roles for minor ailments with sequencing of care through referral that is agreed between pharmacists and general practice for health system efficacy and optimal quality [1,12,35-39]. Specifically, assurance of quality in health service provision may be achieved through the evaluation of standardized condition management and differential diagnosis tools such as *HealthPathways* [40]*,* robust referral processes for escalation, and service delivery by the pharmacist themselves.

In achieving the stated objectives, we may provide evidence that a scheme would be successful in Australia. Community pharmacists offering an enhanced self-care model can make a significant contribution to Australian health care and reduce the substantial burden on other primary care providers with pharmacists providing the appropriate level of care for minor ailments and checking on patients who are self-medicating. The integration of community pharmacists into primary health care would better enable primary care to be delivered in a structured manner. In addition, the systematization of clinical decision making and referrals through relatively easy-to-update protocols would improve service navigation and the patient journey. The development of new clinical pathways in the area of minor ailments seeks to standardize practice according to the best available evidence and reduce variations in current practice. Increased interprofessional teamwork and collaboration between GPs and community pharmacists for care coordination would increase the likelihood of reaching treatment goals and improving patient outcomes. Community pharmacists will gain from having evidence-based guidance, and the community will benefit from another mechanism to ensure that advice from a pharmacist is based on the latest available evidence. AMAS facilitates increased access to care for individuals to receive minor ailment treatment in a timely and efficient manner.

This paper describes a research protocol to evaluate a collaborative protocolized AMAS to improve the management of common ailments in Australia. The AMAS intervention outlined in this study protocol offers a unique and innovative approach to address self-medication and formalize triage processes in the Australian primary care system. The principal aim of this study is to evaluate the clinical, economic, and humanistic impact of AMAS on adult patients attending Australian community pharmacies compared with usual pharmacist care.

## Methods

### Study Design and Setting

The study will use a community pharmacy-based cluster randomized controlled trial (cRCT) design with an intervention group and a control group following the Standard Protocol Items: Recommendations for Interventional Trials checklist [41] (Multimedia Appendix 1). The study will be performed over 8 months in community pharmacies throughout Western Sydney Primary Health Network (WSPHN) region.

### Recruitment of Study Participants

Participant recruitment will occur at 3 levels: community pharmacy, general practice, and patient level.

#### Pharmacy Level

Community pharmacies located in WSPHN region with a pharmacist available to attend specialized training to deliver the AMAS service will be eligible to participate in the study. Contact information of pharmacies will be retrieved from publicly available lists, and those meeting criteria for inclusion will be invited to join the study by telephone. The lead researcher will arrange face-to-face discussion for those expressing interest and to obtain written consent for participation. Randomization will be at the level of the community pharmacy. Pharmacies will be sequentially numbered according to their order of acceptance into the study. An independent researcher will assign the pharmacies (units of randomization) to either the intervention group or control group based on unrestricted random sampling using a computer-generated random number list with a ratio of 1:1 in Excel 2016 (Microsoft Corporation).

#### General Practice Level

Representatives from WSPHN will assist in the engagement and recruitment of general practices within WSPHN into the study. An expression of interest will be forwarded by a blast email to all practices located within the region. The WSPHN representative will provide follow-up information for those expressing interest, and consent will be sought at the practice level from GP practice managers overseeing the work of the surgery or group of surgeries. Each practice manager will be requested to ensure individual GPs within the consented practice are made fully aware of their role within the study before commencement. Study information will be circulated to individual practitioners detailing GP involvement, and given the option of contacting the research team with further questions. Signed practice consent forms will be forwarded to the lead researcher. Informed consent will be essential to receive information from the pharmacist. The details of individual GP involvement in the study are provided below.

#### Patient Level

Patients will be recruited from participating pharmacies. Consecutive recruitment will be used. The recipients of the AMAS service or usual care will be patients who request management for their minor ailment symptoms (symptom-based request) and/or self-select a product to self-treat their ailment (product-based request). The patient may either initiate an interaction or wait to be approached by a member of pharmacy staff while self-selecting a product. The pharmacy team member will refer the patient to the pharmacist who will offer participation in the study if eligible to participate. Patients aged 18 years or older will be identified as eligible if meeting all the qualifying criteria, including (1) attending the pharmacy in person, (2) presenting with a symptom-based and/or product-based request for one of the included minor ailment conditions from 3 specific symptom groups (Table 1), (3) ability to provide written informed consent to participate in the study, and (4) accessible by telephone.

**Figure 1:**
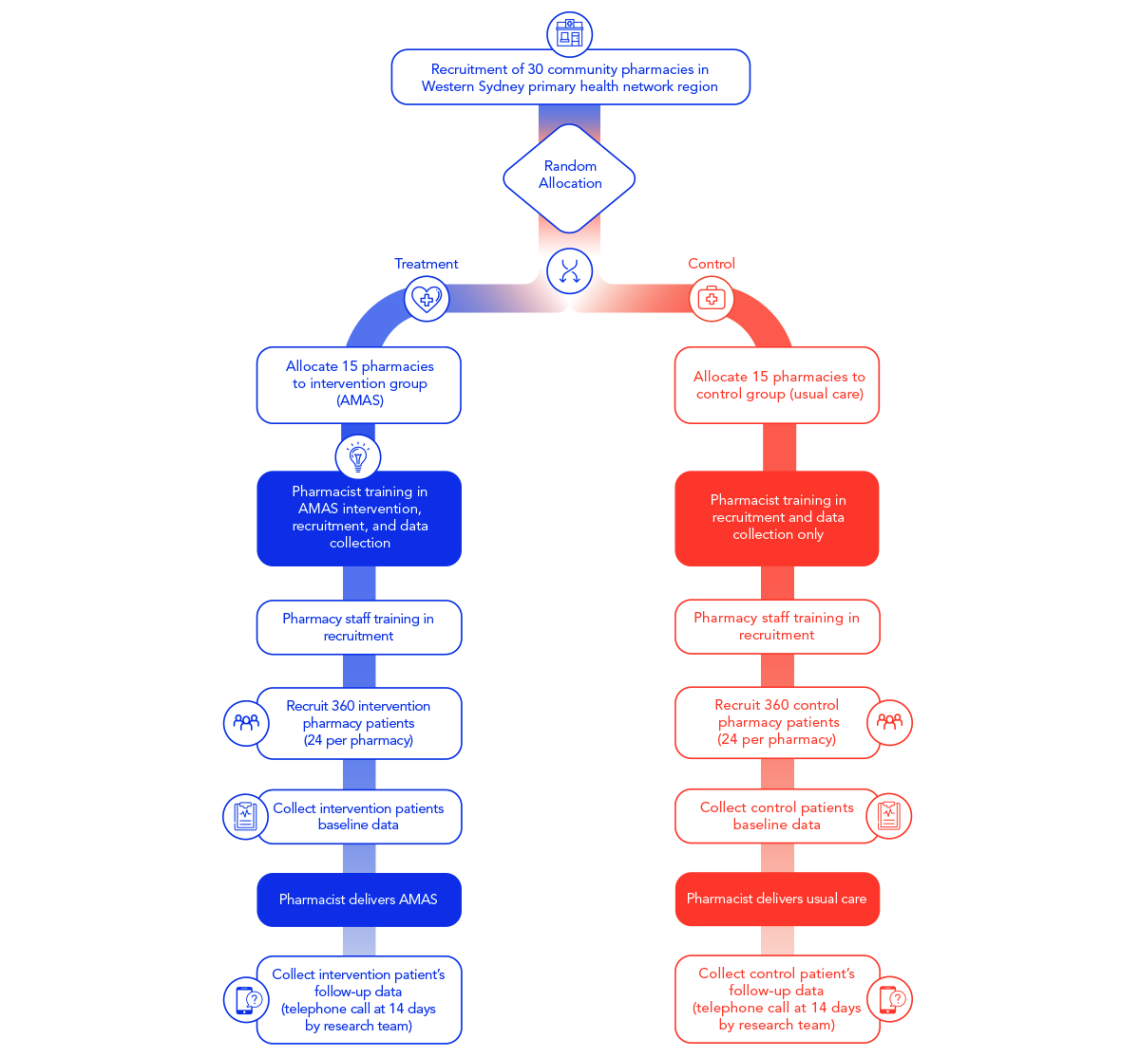
Study design. AMAS: Australian Minor Ailments Service.

Eligible patients identified by the pharmacist will be provided a Participant Information and Consent Form (PICF) explaining the study and given the opportunity to ask questions. Further discussion will be conducted at a private area in the pharmacy or an area appropriate for the discussion to be performed in a confidential manner. Those agreeing to participate will be asked by the pharmacist to provide signed consent. On the basis of which pharmacy they attend, patients will receive the intervention or usual care (Figure 1).

**Table 1:** Minor ailment conditions.

Classification	Minor ailments to be included in the study
Gastrointestinal	Reflux or indigestion
Respiratory	Cough and common cold
Pain	Headache (tension or migraine), primary dysmenorrhea (period pain), and low back pain

### Description of Intervention

**Figure 2:**
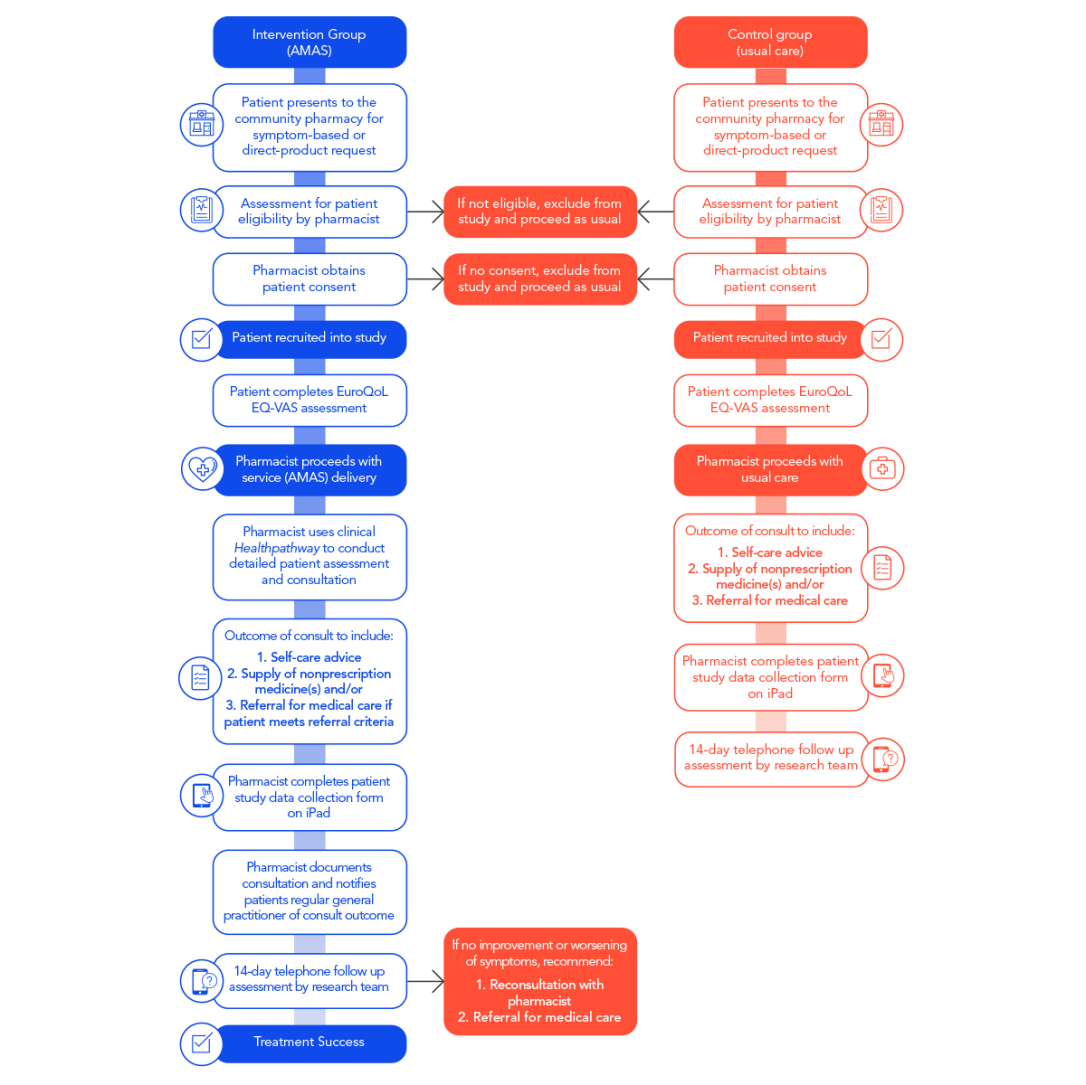
Usual care versus intervention: clinical management algorithm. AMAS: Australian Minor Ailments Service; EQ-VAS: EuroQoL Visual Analogue Scale.

As we are aiming to evaluate the impact of an enhanced service compared with the one that is already being delivered in routine practice, intervention patients will receive AMAS on presentation to the pharmacy. This will involve a protocolized face-to-face pharmacist-patient consultation. Pharmacists will follow a number of steps in the patient encounter (Figure 2). Patients will be followed up at 14 days after the initial patient-pharmacist consultation through telephone by the research team to assess for resolution of symptoms and health care utilization for the same ailment.

We are proposing a number of innovative features to AMAS, which are described below.

#### Collaborative Treatment Pathways for Minor Ailments

Clinical pathways are “document-based tools that provide recommendations, processes, and time frames for the management of specific medical conditions or interventions” [42]. They define a process of care agreed by local clinicians and pharmacists and are informed by existing evidence, guidelines, and protocols. *HealthPathways* is a proprietary system of clinical pathways developed in New Zealand and adopted by clinicians throughout PHNs in Australia [40]. These pathways seek to serve as guidance for desired standards of practice and are ultimately intended to promote consistency and uniformity of care.

The collaborative clinical pathways for each minor ailment (Table 1) are intended for use by community pharmacists delivering AMAS. Each ailment has the same structure and format to make the process of finding and using the information easy and practical. These pathways include types of questions, assessment, management approach recommending a particular course of action including self-care, and/or a nonprescription medicine for symptomatic relief, specific to each ailment. Included is a robust framework for referral, indicating red flag criteria to trigger escalation processes, and the time frame within which a patient is recommended to seek care from a particular health care provider (ie, the patient is recommended to see a GP within 24 hours). A red flag is a symptom that is recognized as likely to be of a more serious nature and requires immediate referral. The research and writing of these clinical pathways followed a literature review of contemporary international and national clinical guidelines in consultation with leading general medical professionals involved in PHN governance with comprehensive experience in *HealthPathways* development.

#### Pharmacist-Directed Care and Data Collection

Pharmacists will undertake a consultation with eligible patients for symptom-based and product-based requests in the community pharmacy. Intervention pharmacists will use the agreed clinical pathways to recommend a particular course of action, including self-care and/or nonprescription medicine recommendation for symptomatic relief and/or referral. In case of the need to refer, the pharmacist will appropriately escalate if the patient meets criteria for referral for further assessment and/or prescribing of prescription-only medicine.

#### Collaborative Approach to Management, Follow-Up, and Data Collection

The *HealthLink* system is used by clinicians in Australia [43]. This system allows for the encrypted transmission of clinical and patient confidential information securely and reliably between GPs and community pharmacists. For AMAS patients who have identified a regular GP during the patient-pharmacist consultation, the consultation will be documented and forwarded from the pharmacist to the GP, outlining clinical assessment undertaken, observations, presentation, and consult outcomes (ie, medication supply, pharmacist-directed self-care, and/or details of referral). Details of the consultation will not be provided if (1) the patient has not consented, (2) the patient has not identified a regular GP, (3) the practice has not consented to partake in the study, or (4) the practice is not using HealthLink software. Importantly, the use of this communication system has been agreed with local clinicians within WSPHN. The process of rolling out this system to pharmacies, set up, and licensing will be facilitated by the PHN and project team. If a patient’s identified GP has not consented to the study or does not use this software in practice, the pharmacist will still provide the AMAS service (ie, following management pathways and referral if required), yet GPs will not receive feedback on details of their patient’s consultation.

#### Training Pharmacists to Deliver Australian Minor Ailments Service

Intervention pharmacists will attend one of two 7.5-hour training workshops at WSPHN before delivery of AMAS. The aim of educational training is to ensure pharmacists competency in delivering the service. The 2016 National Competency Standards Framework for Pharmacists in Australia [44] and the Pharmaceutical Society of Australia’s Professional Practice Standards (version 5) [15] informed the development of content emphasizing competencies to enhance the pharmacist’s role in service provision. The training program will also be a refresher about current best practice in common ailments. The workshop will include a combination of lecture presentations and interactive sessions including role-play scenarios. Self-care information and resources for consumers, clinical treatment pathways, communication and data collection software are available on provided iPads to be used at the point of care. Given that pharmacy assistants are likely to be the very first point of contact in the pharmacy, a researcher will visit each intervention pharmacy to train pharmacy assistants in recruitment and will be given the opportunity to ask questions. During this visit, training materials will be revisited with a *champion* pharmacist who will have attended one of the training days before commencing recruitment.

#### Practice Change Facilitation to Support Intervention Pharmacies

Practice change facilitators (PCFs) will visit intervention pharmacies at least monthly to support the delivery of AMAS. The PCF will be involved in a range of change facilitation processes and activities during visits with the objective of ensuring recruitment targets are met, quality of service provision, quality of data entry, and adherence to the intervention protocol. PCFs will be trained to ensure these objectives are met. These include addressing any barriers to change using evidence-based strategies. PCFs will be collecting both quantitative and qualitative data on-site. This role works closely with the research team.

### Control Group

Pharmacies randomized to the usual care arm will receive training in the use of data collection materials and recruitment only. One training night (2 hours) in data collection and recruitment will be provided at WSPHN. A researcher will visit each of the 15 control pharmacies to deliver study materials, and pharmacists unable to attend the training night will be trained in-store. Materials to be provided include study information detailed in the PICF, data collection software for use on provided iPads, and detailed instructions for data collection. Training will be provided to pharmacy staff to support recruitment for the pharmacist. Patient recruitment will begin immediately after this visit. The pharmacist will check patient eligibility, obtain informed consent, and will document control patients’ baseline data and proceed with usual care using their own clinical judgment, processes, and resources. Patients will be followed up at 14 days after the initial patient-pharmacist consultation by the research team to assess for resolution of symptoms and health care utilization.

### Data Collection Methods

Data will be collected at 2 time points in both intervention and control arms—baseline and 14 days after the consultation. All patients will complete a baseline questionnaire in the pharmacy, including demographic characteristics, and EuroQoL Visual Analogue Scale. Additional data about patient’s ailment history, their contact details, and pharmacist intervention will be collected by pharmacists using forms on iPads provided for that purpose. The time taken per patient to deliver the intervention or usual care will be recorded to inform the economic analysis. Follow-up telephone questionnaires will be conducted by research assistants using forms provided for that purpose. Follow-up at 14-days is considered appropriate because of the nature and duration of minor health symptoms. Study data will be collected and managed using Research Electronic Data Capture (REDCap) tools hosted at the University of Technology Sydney (UTS) [45]. REDCap is a secure, web-based application designed to support data capture for research studies, providing (1) an interface for validated data entry, (2) audit trails for tracking data manipulation and export procedures, (3) automated export procedures for data downloads to statistical packages, and (4) procedures for importing data from external sources [45]. All data collected in pharmacies will be returned to the research team on the day of recruitment to allow for timely follow-up. The chief investigator will have access to the trial data.

### Study Measurements and Outcomes

The evaluation of MAS compared with usual care will be achieved by comparing the primary and secondary outcomes [46] as set out in Multimedia Appendix 2.

### Sample Size

The primary joint outcome measures of the study are appropriate medical referral rate and appropriate recommendation of nonprescription medicines. Sample size calculation was based on an assumed baseline appropriate medical referral rate of 85% and assumed baseline appropriate recommendation of nonprescription medicines rate of 82% [47,48]. Pharmacies are the primary unit of randomization with individual patients nested within pharmacies. The rate of the joint outcomes will be compared between the treatment and control arms in the study. To test for a 10% absolute increase in primary outcomes (appropriate medical referral rate: 85%-95% and appropriate recommendation of nonprescription products 82%-92%) with ≥0.9 power, alpha of .05, equal allocation ratio, and assuming intracluster correlation is 0.01, we would need 30 pharmacies (15 in each arm) with 24 participants per pharmacy (allowing for 10% dropout) for an overall sample of 720 patients.

### Blinding

Given the cluster design, it will not be possible for participating pharmacies to be blinded to group assignment. However, the patient, research assistants conducting follow-up, and the data analyst will be blinded to treatment assignment.

### Postrecruitment Retention Strategies

All recruited pharmacies will be contacted by telephone in the first 2 weeks of commencing patient recruitment to address any teething issues with study procedures. Support to resolve any problems will be offered by PCFs (for intervention) or a study researcher (for control). Intervention fidelity will also be monitored by PCFs. Regular newsletters and emails will be sent to all pharmacies during the study period for encouragement, provision of feedback surrounding data quality, and strategies to enhance recruitment to meet desired targets. Pharmacies not meeting target recruitment will be offered additional in-pharmacy support by the study researcher. Recruited patients will be contacted by telephone. Attempts to contact nonresponders will continue until contact is made or for a maximum of either 1 week or 5 call attempts.

### Statistical Methods and Analysis

Data will be analyzed using Stata 16 for Windows [49]. Baseline pharmacy and patient level information will be summarized by treatment arm. Continuous variables will be summarized with mean and standard deviation with median and interquartile range provided if the data are skewed. Categorical variables will be summarized by frequency and proportion. Generalized estimating equations will be used to account for within-cluster correlation [50] using an exchangeable correlation structure. A modified Poisson regression approach will be used for the analysis to estimate relative rates (RRs) [51,52]. If the estimation of RR is not computationally achievable, we will estimate odds ratios with logistic regression [50]. As a secondary analysis, we will adjust for key baseline covariates at both the pharmacy level (eg, pharmacy type) and the patient level (eg, age and sex). We plan to conduct an exploratory subgroup analysis by treatment classification (respiratory, pain, and gastrointestinal) and type of inquiry (symptom presentation, direct product request, and both). Standard model diagnostics will be conducted to check for model assumptions. All analyses will be intention-to-treat. Multiple imputation (MI) by chained equations [53] will be applied to account for missing patient outcomes. A total of 30 imputations (including using pharmacy type, age, and sex in the MI model) will be performed. A detailed statistical analysis plan will be developed by blinded investigators before unblinding and locking the study database.

A cost-utility analysis (CUA) and cost-effectiveness analysis (CEA) will be performed through examining the resource use of adult patients in the context of the randomized controlled study designed to investigate the efficacy of AMAS compared with the control group. A healthcare perspective will be applied for the analysis. Costs will be estimated in Australian dollars at the 2018-2019 financial year. Costs during the 2-week follow-up period will be analyzed for all patients included in the cRCT. Costs will be grouped into 4 main categories: (1) pharmacist time, (2) medications, (3) referrals and reconsultation, and (4) training and facilitation costs. The pharmacist cost will consider the working time for a community pharmacist and time consumption to deliver the service. Patient out-of-pocket costs (for all medicines supplied during the 14-day period) will be estimated by the average unit price across pharmacy banner groups. Health service utilization will be based on the cost of medical services recorded in the study, with unit prices sourced from Medicare Benefits Schedule prices, Australian National Hospital Cost Data [54], and the Pharmacy Industry Award [55]. Finally, capital costs for training of pharmacists, facilitation, information technology, and program setup will be counted.

The trial-based outcome measures used for the economic evaluation will be symptom resolution rates and appropriateness of pharmacy care (as a proxy of health gain). Utility values from the literature for symptom resolution and nonsymptom resolution of minor ailments will be used to estimate quality-adjusted life years (QALYs). Other intermediate outcomes will be used to adjust the utilization of resources including referral and reconsultation rates. A decision analytic modeling technique will be used. The model inputs will be informed by data from the trial supplemented with published literature. Results of the CUA will be expressed in terms of an incremental cost per QALY (incremental cost-effectiveness ratio), calculated by dividing the difference in total costs and QALYs between intervention and control groups (incremental costs/incremental QALYs). In addition to the CUA, 2 CEAs will be conducted where the clinical effect measure will be an extra episode of appropriate pharmacy care and extra patient achieving symptom resolution for their ailment. The cost-effectiveness results will be expressed in terms of extra cost per additional episode of appropriate pharmacy care and extra cost per additional patient achieving symptom resolution.

### Ethics Approval and Consent to Participate

This project has been approved by the UTS Human Research Ethics Committee (HREC) (UTS HREC approval number: ETH17-1350). All participants (pharmacies, general practices, and patients) will complete a consent form to participate in this research.

## Results

Statistical and economic analyses will be completed in July 2019. Following this, research findings will be disseminated through peer-reviewed publication.

## Discussion

### Integrated Care

Globally, health care is changing to address a number of challenges including the needs of an aging population, escalation in consumer knowledge and their expectations of the health service, rapid advances in scientific and technical capacity, and the increasing cost of health care [56]. With this, a key issue that needs to be addressed is how to connect services and health care professionals to achieve integrated services for consumers and health professionals as models of care evolve to deliver a person-centered approach [57]. There are excellent services and health professionals all striving to deliver the best possible care, but it is often in a fragmented and siloed manner [2]. The increasing longitudinal care requires both effective oral and *technology-enabled* communication between health care team members.

Innovative thinking and tools are needed to deliver better and cost-effective care. This study is unique, as it enables and evaluates integrated electronic technology systems in Australian primary care for common ailments. This ensures health care providers have access to the best information available to deliver excellent patient care. Although the journey to integrated care is complex, technology can help to support it; this applies to care management and referral (*HealthPathways* [40]), collection of data (*REDCap* [45]), and interprofessional clinician-pharmacist communication (*HealthLink Messaging Software* [43]). This approach offers innovative technologies to move from the traditional health care delivery model, which centers on individual disciplines operating in isolation, to solutions that integrate systems to provide a centralized, complete patient view to health care providers.

This research supports an integrated approach in managing common minor ailments. Drawing on expertise from a range of stakeholders, an AMAS service has been co-designed to complement general practice and promotes collaboration between professions. With the development of agreed clinical *HealthPathways* for a number of common ailments [40], the service aims to standardize practice according to the best available evidence and reduce variations in current practice using a robust framework for referral and treatment. To our knowledge, there is no study investigation or published research relating to a protocolized MAS intervention delivered by community pharmacists for minor ailment presentations in Australian health care. This research will evaluate an Australian MAS reporting on patient outcomes, including health status, and resolution of symptoms and will provide full economic analyses. This evaluation focuses on specific minor ailments for relevant comparisons of both health-related and cost-related outcomes.

### Comparison With Literature

The literature internationally suggests that minor ailment services enhance the delivery of primary care, promote efficiencies, and reduce overall health care costs [20]. Pharmacy-based minor ailment services were introduced internationally over a decade ago with the aim of supporting consumers to self-care and provide professional support for conditions that can be self-managed [20]. Previous evidence includes the studies by Paudyal et al [21], Watson et al [16], Aly et al [20], and Rafferty et al [58] reporting on minor ailment services. From the UK perspective, studies have compared outcomes of minor ailment management in settings such as pharmacy, emergency departments (EDs), and general practice [16]. The positive economic impact of MAS has been demonstrated through reduced pressure on other health services and cost-effectiveness compared with more expensive health care services, such as general practice and A&E [16]. Comparatively, Rafferty et al have identified community pharmacy as the most cost-effective option for minor ailment care in Saskatchewan, Canada [58]. The scope of complexity and the varied nature of conditions treated by pharmacists under MASs highlight their skills in being able to assist consumers to self-care, facilitating self-medication, ensuring appropriate use of medicines, and timely medical referral [20]. Comparative evaluations identified in the literature compare general practice or ED settings to the community pharmacy or interventions delivered by health care professionals in ED and GP (ie, physicians or nurses) as a comparator to community pharmacy-based MAS [16,59,60]. Within the various studies, there is no clear distinction between whether pharmacists or members of pharmacy staff deliver the MAS intervention. Our study delineates the role of pharmacist in delivering the MAS intervention, and is not delivered by support staff under pharmacist supervision in the pharmacy.

We report 2 primary outcome measures (appropriate medical referral and appropriate recommendation of nonprescription medicine by pharmacist). Referrals (and importantly, red flag referrals) were a critical point that came up in the codesign process with GPs. GPs wanted to see patients quickly if there were any doubts and ensure patients are being referred in an appropriate and timely manner to the correct health provider. We also wanted to assess pharmacist’s impact of MAS on self-medication processes. Further strengths to the study include the adoption of clinical and humanistic outcomes (as secondary outcome measures) recommended by Paudyal et al in a systematic review published in 2018 [61]. Clinical outcomes identified in this international review included symptom status (such as resolution of symptoms, symptom severity, and pattern). Reconsultation with the GP was identified as a surrogate follow-up measure of clinical outcome assessment. Our study will evaluate reconsultation with the pharmacist, GP, and other health professionals within 14 days for the same ailment. Quality of life outcomes using EuroQoL have also been previously collected in a number of studies [61,62]. Our intervention was developed using available evidence and theory, with key elements. Methods of recruitment, data collection, and study variables were tested during a feasibility and piloting stage. This helped to identify methods to improve recruitment rate, limit documentation time, and confirm relevance and appropriateness of study outcomes to Australian health care.

We present the design of a cRCT in international literature to determine the clinical, humanistic, and economic effectiveness of a protocolized intervention for minor ailments compared with usual care. This study improves on other research evaluating MAS directly using a randomized study design. The randomized controlled trial has a number of important features that make it the *gold-standard* evaluation method [63]. Our choice of cluster randomization at the level of the pharmacy decreases the potential for contamination, as each pharmacist in either the intervention group or the control group will only be providing either AMAS or control, not both. In this respect, the study is novel and will provide information on the impact of the service on clinical, economic, and humanistic outcomes and barriers to implementation compared with usual pharmacy care. However, some limitations to the study should be discussed. Although a cluster randomized design is being used to overcome contamination between study arms, the study design may be susceptible to some methodological biases. Cluster randomized trials often do not, or cannot, conceal treatment allocation. Participants awareness of the allocation can lead to biased recruitment [63]. The Hawthorne effect may also influence research subjects, that is, the consequent effect of being observed or awareness of being studied which can potentially impact on participants’ behavior [63]. Finally, one of the main limitations of this type of study is that, by definition, a minor ailment is a self-limiting health problem and implicitly involves resolution, regardless of the intervention performed by the pharmacist. Careful attention has been placed to the design of our cluster trial to minimize the potential for biases.

### Conclusions

Collectively, the findings from this study will act as the first stage of implementation of MAS in Australian pharmacy practice and may be extended to facilitate the growing prominence of self-care. The study may also provide groundwork for the optimal design of a MAS intervention tailored for greater patient autonomy and boost the clinician-pharmacist relationship for greater discussion surrounding both the appropriate and inappropriate use of nonprescription medicines. This study evaluates the best possible care to the current level of care provided by pharmacists to patients with common ailments in the Australian population. AMAS presents a key opportunity for pharmacists to intervene, as communication of patient-centric clinical information between health care providers will be essential to support effective patient management in Australian health care.

The delivery of safe and high-quality health services that are fully integrated into the health system are of high importance. Research from high-quality evaluations should be used to inform the strategic direction for health service delivery internationally. Implementation research may be applied to MAS to translate evaluation findings into practice for meaningful improvements in patient care outcomes. This paper is a key step in the dissemination process, outlining the aims and methodology that will be used. Along with providing community pharmacists a framework to patient management and the practical skills to engage patients to self-care and self-medicate appropriately, this study may also contribute to the literature with evidence that an intervention of this nature may lead to more efficient resource use in the provision of primary health care in Australia.

### Dissemination Plan

To support this study's contribution to wider knowledge, the research findings will be disseminated through peer-reviewed publications and conferences, both nationally and internationally, targeting service users, health care providers, academics, service commissioners, and policymakers.

### Trial Status

The study began in July 2018. A total of 30 community pharmacies were recruited. Pharmacists from the 15 intervention pharmacies were trained. 27 general practices consented. Patient recruitment began in August 2018 and was completed on March 31, 2019.

### Protocol Amendments

Any protocol amendments will be submitted to the UTS HREC for approval and noted in the registered protocol at the Australian New Zealand Clinical Trials Registry. Trial participants will be notified should relevant protocol changes be made.

## Appendix

Multimedia Appendix 1 Standard Protocol Items: Recommendations for Interventional Trials checklist.

Multimedia Appendix 2 Summary of measurements and study outcomes.

## Abbreviations

A&E: accident and emergency
AMAS: Australian Minor Ailments Service
CRCT: cluster randomized controlled trial
CEA: cost-effectiveness analysis
CUA: cost-utility analysis
ED: emergency department
GPs: general practitioners
HREC: Human Research Ethics Committee
MAS: minor ailment schemes
MI: multiple imputation
PCF: practice change facilitator
PHN: primary health network
PICF: Participant Information and Consent Form
QALYs: quality-adjusted life years
REDCap: Research Electronic Data Capture
RR: relative rate
UTS: University of Technology Sydney
WSPHN: Western Sydney Primary Health Network
