# Supplementary material for: Evaluation of a Collaborative Protocolized Approach by Community Pharmacists and General Medical Practitioners for an Australian Minor Ailments Scheme: Protocol for a Cluster Randomized Controlled Trial
Source: JMIR Res Protoc. 2019 Aug 9;8(8):e13973. doi: 10.2196/13973 (PMC6709938; doi:10.2196/13973)
Supplement: Multimedia Appendix 2 [file resprot_v8i8e13973_app2.pdf]

Variable	Operational definition	Group	Type	T0[a]	T2[b]	Data source	Completed by
**Clinical**							
Appropriate medical referral rate	Defined as meeting the action agreed in the HealthPathways for each patient referred. Each referral made will be independently assessed against the action outlined within the HealthPathways for each minor ailment indication (which were preagreed with GPs[c] in the codesign process). The referral is considered appropriate if it meets the reason for referral, recommended time frame to seek care, and health care provider referred to. In which case, the appropriateness of referral will be calculated as the proportion of patients appropriately referred divided by the total number of patients referred for treatment and control arms.	IG[d], UG[e]	1°[f]	X	—[g]	Patient consultation record	Pharmacist
Appropriate recommendation of nonprescription medicine rate	Defined as meeting the action agreed in the HealthPathways for each product recommended. Each product recommendation will be independently assessed against the action outlined within the HealthPathways for each minor ailment indication (which were preagreed with GPs in the codesign process). The recommendation is considered appropriate if it meets the entire requirement as approved in Product Information by the Therapeutic Goods Administration including correct indication for use, dose, frequency, duration of use, and contraindications. The appropriateness of	IG, UG	1°	X	—	Patient consultation record	Pharmacist

	medicine recommendation will be calculated as the proportion of patients receiving an appropriate medicine recommendation by the pharmacist divided by the total number of patients who received a medicine during the consult for treatment and control arms.						
Pharmacist intervention rate (or clinical intervention rate) for direct product requests	Defined as the identification and attempted resolution of an actual or potential drug-related or symptom-related problem arising from a patient self-selecting a medicine to self-treat. An investigation of the pharmacist’s identification and response (ie, change in product to a safer or more appropriate alternative) will be made. In which case, the clinical intervention rate will be calculated as the proportion of patients recommended an alternative product by the pharmacist divided by the total number of patients who present to the pharmacy directly requesting a product for self-treatment for treatment and control arms.	IG, UG	1°	X	—	Patient consultation record	Pharmacist
Self-reported symptom resolution rate	Participants will be asked at follow-up to indicate whether their minor ailment symptoms have (1) completely resolved, (2) improved but not completely resolved, and (3) not improved or have worsened. Complete resolution has been defined as the complete absence of minor ailment symptoms at 14-day follow up. In which case, the symptom resolution rate will be calculated as the proportion of patients reporting complete symptom	IG, UG	$2^{\circ h}$	—	X	Telephone data collection record	Research team member

	resolution at 14-day follow-up divided by the total number of patients successfully followed up for treatment and control arms.						
**Economic**							
Health services resource utilization associated with the minor ailment	Defined as the individual's use of pharmaceutical, GP, hospital, and emergency department services within 14 days following the initial consultation with the pharmacist for treatment and control arms.	IG, UG	2˚	X	X	Patient consultation record, telephone data collection record	Pharmacist and research team member
Time and resources of service delivery	Defined as the time and personnel consumptions for AMAS[i] delivery and usual care.	IG, UG	2˚	X	X	Patient consultation record, facilitators database	Pharmacist, research team member, and practice change facilitator
**Humanistic**							
Change in self-reported EQ-VAS[j]	Defined as patient's overall measure of health status at (1) the initial consultation with the pharmacist and (2) 14 days following the initial consultation with the pharmacist for treatment and control arms.	IG, UG	2˚	X	X	EuroQoL Visual Analogue Scale [46]	Patient

[a]T0: baseline.

[b]T2: follow-up at 14 days.

[c]GP: general practitioner.

[d]IG: intervention group.

[e]UG: usual care group.

[f]1˚: primary outcome.

[g]—: not applicable.

[h]2˚: secondary outcome.

[i]AMAS: Australian Minor Ailments Service.

[j]EQ-VAS: EuroQoL Visual Analogue Scale.
